# A Quick Guide to Teaching R Programming to Computational Biology Students

**DOI:** 10.1371/journal.pcbi.1000482

**Published:** 2009-08-28

**Authors:** Stephen J. Eglen

**Affiliations:** Cambridge Computational Biology Institute, Department of Applied Mathematics and Theoretical Physics, University of Cambridge, Cambridge, United Kingdom; Whitehead Institute, United States of America

## Introduction: Why Use R in Computational Biology?

The name “R” refers to the computational environment initially created by Robert Gentleman and Robert Ihaka, similar in nature to the “S” statistical environment developed at Bell Laboratories (http://www.r-project.org/about.html) [1]. It has since been developed and maintained by a strong team of core developers (R-core), who are renowned researchers in computational disciplines. R has gained wide acceptance as a reliable and powerful modern computational environment for statistical computing and visualisation, and is now used in many areas of scientific computation. R is free software, released under the GNU General Public License; this means anyone can see all its source code, and there are no restrictive, costly licensing arrangements. One of the main reasons that computational biologists use R is the Bioconductor project (http://www.bioconductor.org), which is a set of packages for R to analyse genomic data. These packages have, in many cases, been provided by researchers to complement descriptions of algorithms in journal articles. Many computational biologists regard R and Bioconductor as fundamental tools for their research. R is a modern, functional programming language that allows for rapid development of ideas, together with object-oriented features for rigorous software development. The rich set of inbuilt functions makes it ideal for high-volume analysis or statistical simulations, and the packaging system means that code provided by others can easily be shared. Finally, it generates high-quality graphical output so that all stages of a study, from modelling/analysis to publication, can be undertaken within R. For detailed discussion of the merits of R in computational biology, see [2].

## How to Teach R to Students

This brief article is an introduction to teaching R, based on my experience in teaching computational biology graduate students. R is a powerful environment for teaching many aspects of computational biology, including functional genomics, computational neuroscience, dynamical systems, statistical genetics, and network biology. I provide resources and suggestions for teaching R and describe common difficulties faced by students when learning R.

### Lecture material

Most students starting our master's programme have not previously seen R; at first, we assumed that students would self-learn R during the course. However, this proved to be unsatisfactory, as students often said that R was too difficult to learn on their own on top of their assigned coursework. In response to this problem, we created an intensive set of lectures and lab sessions covering both an introduction to programming in R and a refresher in statistics (the introductory statistics material is not covered here).

Given that students come from different backgrounds, some with experiences of programming in other languages and others without any prior programming experience, it is difficult to know at exactly which level to aim a lecture course. Our approach has been to provide lecture material containing concepts that may not immediately be understood by novice programmers, but will serve as a reference for them later in the year. Instead, the lectures contain advanced material that can challenge students who have programming experience. Our lecture notes are available in Text S1. The Bioconductor project also offers useful teaching material (http://www.bioconductor.org/workshops). A key aim when writing these lecture notes was to focus on teaching R as a general programming language, rather than to focus on issues specific to computational biology. Other lecture courses on our master's programme provide additional R material relevant to particular topics in computational biology.

### Assignments/lab sessions

The best way that the students learn a programming language is by actually using the language on problem sets. We therefore arrange lab sessions during which students work through introductory material on R. After becoming familiar with R, we then suggest they work on some problems in computational biology. Good sources for such problems include [3], as well as the R guide to accompany [4], described below. We also suggest that students read descriptions of various popular problems and then implement them in R. These include:

- Sequence alignment. A basic algorithm is concisely described by [5].
- The discrete logistic equation [6]. Students are asked to generate a bifurcation diagram showing how the steady state of the logistic equation varies as a key parameter varies.
- Cellular automata. Conway's game of life [7] provides a nice example of studying cellular automata.

As well as learning about particular concepts useful in computational biology (e.g., difference equations, dynamic programming), these exercises test students' abilities in vector and matrix manipulation, looping, conditionals, file input/output, and plotting.

### Using R for generating graphs

R generates high-quality graphical output. It is worth providing simple examples for generating graphs that can be used as templates (as given in the lecture notes) for their work. Students often fail to realise the difference between vector and bitmap formats, and this is worth discussing in class to suggest they generate graphs using either PDF or Postscript devices, rather than bitmap formats. R currently has two systems for generating graphs: “base” and “grid”. The base system is much simpler and easier to use, and so we recommend students learn this system (and most introductory books and resources also use base graphics). However, students should be made aware of the grid package, which allows for much more flexibility over generating graphics. In particular, the lattice package [8] uses the grid package to allow the user to quickly generate sophisticated and flexible graphics.

### Reproducible research

The idea of reproducible research is quite simple: to provide not only a brief description of, e.g., how some data has been analysed, but also to provide the code and data to allow someone else to recreate exactly the same sequence of steps [9]. R provides infrastructure for this in the form of Sweave documents. Sweave documents contain R code surrounded by documentation written in either LaTeX, HTML, or OpenOffice Writer. The document is processed to extract and run the R code; output (either textual or graphical) is then inserted back into the document which is then typeset. An example of this is shown in Supporting Information files Text S2 and Text S3 (estimating the value of 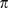). Students should be taught about the idea of reproducible research, and the idea should be reinforced by asking them to submit their coursework in the form of Sweave documents. Reproducible research also encourages students to run their code in batch mode (whereas most students initially prefer working interactively with R). As students need to know LaTeX to write Sweave documents, we also provide a separate lab session on LaTeX. Larger pieces of reproducible research are likely to be released in an R package (containing both data and code), but teaching students how to build packages is beyond the scope of our current course. Writing Sweave documents takes much longer than writing R scripts, but it leads to self-documenting work that is likely to be understandable by many researchers long after it has been written.

### Textbooks

The R website currently lists over 80 books, together with short descriptions that may help the reader decide which books to select (http://www.r-project.org/doc/bib/R-books.html). Here I give a short, non-exhaustive list of books that I recommend to students to complement lecture notes and to show applications of R in computational biology. Some of the books are quite advanced and are likely to be useful for students only after they have gained sufficient experience. I also take these books to lab sessions so that students can see which book would be most useful for them.

For a general introduction to R, *Introductory Statistics with R* [10] provides a nice balance of introducing R and showing its application to classical statistical testing; *Introduction to Probability with R* [11] goes further into aspects of probability. *A First Course in Statistical Programming with R* [12] introduces R as a programming language; those already familiar with programming may wish to consult *S Programming* [13]. Finally, for students wishing to explore the graphing facilities of R, *R Graphics* [14] is recommended.

Several texts focus on aspects of computational biology. First, the introductory text on *Computational Genome Analysis* [3] provides worked examples in R throughout the book. *Stochastic Modelling for Systems Biology* [15] uses R to demonstrate modelling in systems biology. An advanced book for those already familiar with R is *R Programming for Bioinformatics* [16]. Finally, a general text for biological modelling is *Dynamical Models in Biology* [4]. Although the book does not describe R, the online supplementary information provides a comprehensive introduction to R and shows how to use R to simulate the models discussed in the book, along with numerous exercises (http://www.cam.cornell.edu/~dmb/DMBsupplements.html).

### Useful web sites

R has numerous online resources that students should be encouraged to explore. Here are some additional sites that we have found useful:

http://www.rseek.org. Powered by Google, this site searches numerous online R resources, including documentation, source code, and books. It also searches the numerous email lists hosted by the R project; R-help in particular is a useful list for people to learn about R.

http://germain.its.maine.edu/~hiebeler/comp/matlabR.html. A very useful guide for students who know Matlab; it provides a comprehensive list of Matlab functions and the corresponding functions in R.

http://addictedtor.free.fr/graphiques/. This site provides a gallery of advanced graphic examples, along with the R code used to generate those plots.

### Common problems encountered when learning R

Students with previous programming experience usually find learning R quite straightforward. It has a rich set of online documentation for each function, complete with examples, to help learn the language. However, there are some common problems that occur when learning R, described briefly below, along with suggestions for helping students.

#### Syntax errors and getting started

The syntax of R can be difficult for students to acquire, and students often report that they spend many hours debugging simple problems. We encourage students to ask a colleague for help, as often these errors are simple, yet frustrating to spot. We use a wiki to allow students to post questions or exchange tips and example code. Furthermore, although R has a rich set of documentation for inbuilt functions, students often report that it is hard to discover these functions, as they do not know what to search for. With this in mind, our introductory lecture notes were written to describe most core R functions with which we would expect a student eventually to become familiar during the year. Of course, it is infeasible to provide a complete list, especially given the vast number of numerical routines that come with R, and for this we suggest using the Rseek internet search tool, described above. Lecturers should also give hints as to which functions might be of use for particular assignments.

#### Pre-allocation of variables

In R, variables do not need to be defined before use; they are simply created when required. A common problem with this is demonstrated in the following code:

npts <− 100

x <− runif(n = npts)

y <− 0

for (i in 1:npts) {

 if (x[i]>0.5)

  y[i] <− 1

 else

  y[i] <− 0

}

A vector x of 100 random values is generated from a uniform distribution; each element of the vector y should be “one” if the corresponding element of x is greater than 0.5, and “zero” otherwise. A key problem here is that on line 3, y has been initialised to be the value zero, which is a vector of length one. Within each cycle of the loop, the length of y needs to increase by one, and so R silently reallocates the vector y to be long enough to store the new result. The code works, but is inefficient, especially when looping over many values. A simple solution is to pre-allocate the vector when the length of the vector is known in advance. In this case, we can change line 3 to read:

y <− rep(0, npts)

#### Vectorization

Many operations in R process entire vectors at once. For example, if x and y are vectors of the same length, then z = x+y will create a vector z, where for each element i, z[i] <− x[i]+y[i]. This is called vectorization, and students familiar with other programming languages, such as C, often use slow and inefficient for loops to perform these calculations. It is worth reminding students at several stages while they are learning R that they should try to think about how to vectorize their code. Sometimes this requires them to learn new R functions, such as the apply family of commands. For example, if we wish to compute the mean of each column of a matrix mat, rather than writing an explicit loop over each column, we can do:

x <− apply(mat, 2, mean)

The apply family of functions are powerful, but require careful explanation of how they work. In particular, it should be explained that R is a functional language and hence “everything is an object”, which is why functions, such as mean above, can be passed as arguments to other functions.

Continuing the example in the previous section, at first glance it may not seem suitable for vectorizing, given the if-then test operating on each element. However, R has the function ifelse, which simplifies the threshold example to:

npts <− 100

x <− runif(n = npts)

y <− ifelse(x>0.5, 1, 0)

In this case, as well as avoiding the for loop, the problem about allocating the size of the resulting vector y has gone. Vectorized solutions are often shorter, too, implying that there is less code to maintain.

Even when students are familiar with vectorization, a common question asked is how to recognise which code might benefit from vectorization. The answer, unfortunately, is that it requires accumulating experience at applying various tricks. Students can be helped by giving them examples, such as the one in the following paragraph, and asked to study it so that they understand exactly how it works. Warnings should be given, however, that even simple problems, such as computing the Fibonacci series, are impossible to vectorize. It is better to get the code working correctly and then worry about efficiency later: “…premature optimization is the root of all evil (or at least most of it) in programming… [17]” (on the other hand, even when a correct R program is optimised, it may still be too slow, in which case the compute-intensive parts can be rewritten in C and called from R).

#### Example vectorization problem

Given a vector of event times e, write a function to return the interval between successive events, e.g., interval[i] <− e[i+1]−e[i]. Solution: Given that the vector e and the result are of different lengths, it may seem that vectorized solutions are not possible. However, by using the “minus indexing” notation (e[−j] returns a vector with everything except element j of e), we can easily vectorize the problem:

diff1 <− function(e) {

 ## Explicit loop

 n <− length(e)

 interval <− rep(0, n−1) ## pre-allocate result

 for (i in 1:(n−1)) {

  interval[i] <− e[i+1]−e[i]

 }

 interval

}

diff2 <− function(e) {

 ## Vectorized solution

 n <− length(e)

 e[−1]−e[−n]

 }

x <− c(5.9, 10.2, 12.4, 18.8)

all.equal(diff1(x), diff2(x))

#### Data types

A common concern raised by students is that they are not sure when to use the different data types (e.g., list, data frame, matrix) to store their data or how to convert from one type to another. Part of the problem is caused by the flexibility in R for functions to transparently handle different data types. Again, such issues normally resolve themselves by continued exposure to R, but instructors can help by showing how the type of an object can be determined and how objects can be converted from one type to another. Relationships among data types should also be highlighted (e.g., a matrix being a particular kind of vector, and a data frame being a particular kind of list).

## Closing Comments

In this article I have summarised our experience to date on teaching R. As the last section has shown, there are several difficulties with learning R, but I believe that they are fairly minor compared to the benefits in using such a powerful environment. Learning R is an ongoing process, and once students have mastered the basics, they should be encouraged to explore the wealth of contributed packages on the Comprehensive R Archive Network (CRAN) (http://cran.r-project.org) and Bioconductor (http://www.bioconductor.org).

## Supporting Information

Text S1 Lecture notes for programming in R.
(0.48 MB PDF)

Text S2 Example Sweave document demonstrating how R code and LaTeX code can be combined.
(0.00 MB TXT)

Text S3 PDF output from the example Sweave document.
(0.11 MB PDF)
