# Supplementary material for: A Quick Guide to Teaching R Programming to Computational Biology Students
Source: PLoS Comput Biol. 2009 Aug 28;5(8):e1000482. doi: 10.1371/journal.pcbi.1000482 (PMC2725315; doi:10.1371/journal.pcbi.1000482)
Supplement: Text S1 — Lecture notes for programming in R. (0.48 MB PDF) [file pcbi.1000482.s001.pdf]

R Programming

Stephen Eglen

June 9, 2009

Books and online help

- Introductory Statistics with R (Springer, Dalgaard).
- A first course in statistical programming with R (CUP, Braun and Murdoch).
- Computational Genome Analysis: An Introduction (Springer, Deonier, Tavaré and Waterman).
- S programming (Springer, Venables and Ripley).
- R programming for Bioinformatics (CRC Press,Gentleman).
- Writing Scientific Software (WSS) (CUP, Oliveira and Stewart).
- `www.r-project.org`, `www.rseek.org`
- R-help mailing list.

Aims of course

This course aims to teach R as a general-purpose programming language. Issues specific to Computational Biology (e.g. Bioconductor packages) are covered in other course modules.
Topics to be mastered in this course include:

- Interactive use of R.
- Basic data types: vector, matrix, list, data.frame, factor, character.
- Writing scripts.
- Graphical facilities.
- Writing your own functions.
- File input/output.
- Control-flow statements, looping.
- Vectorization.
- Numerics issues.
- Debugging.

What is R?

- Computing environment, similar to matlab.
- Very popular in many areas of statistics, computational biology.
- "Programming with data" (Chambers)
- Approach: command-line for one-liners; write scripts/functions for larger work (edit/run cycle).

History

- S language came from Bell Labs (Becker, Chambers and Wilks). Commercial version S-plus (1988).
- R emerged as a combination of S and Scheme: Ross Ihaka and Robert Gentleman (NZ).
- 1993: first announcement.
- 1995: 0.60 release, now under GPL.
- Oct 2008: release 2.7.2. Stable, multi-platform. Major release typically Apr/Oct with fixes between. (2.8.0 due 2008-10-20).
- R-core now 20 people, key academics in field, including John Chambers.

Strengths of R

- GPL'd, available on many platforms.
- Excellent development team with Apr/Oct release cycle.
- Source always available to examine/edit.
- Fast for vectorized calculations.
- Foreign-language interface (C/Fortran) when speed crucial, or for interfacing with existing code..
- Good collection of numerical/statistical routines.
- Comprehensive R Archive Network (CRAN) $\sim$ 1550 packages.
- On-line doc, with examples.
- High-quality graphics (pdf, postscript, quartz, x11, bitmaps). Often used just for plotting ...

Graphics example

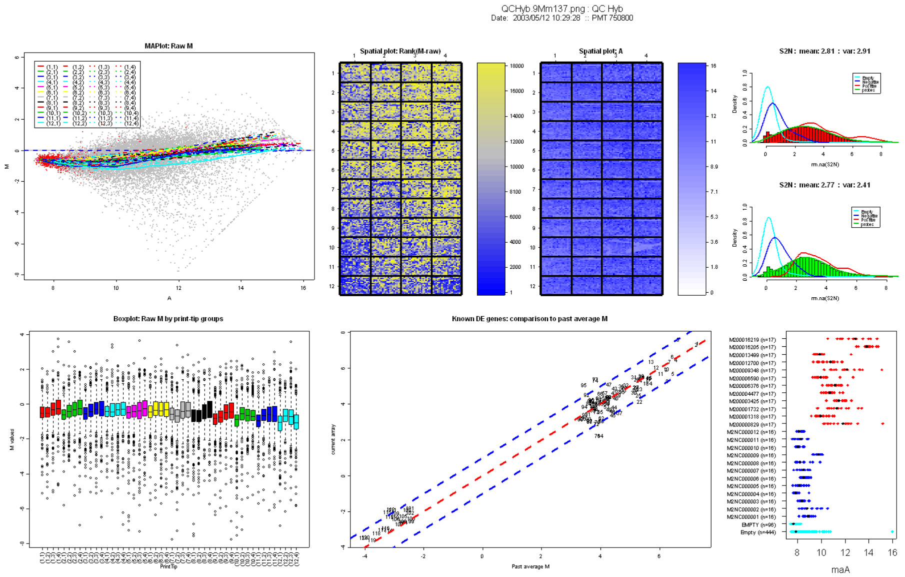

Jean YH Yang; gpQuality
`http://bioinf.wehi.edu.au/marray/ibc2004/lect1b-quality.pdf`

Weaknesses of R

- Loops are slow. Learn how to vectorize solutions or use apply family of functions.
- No compiler yet, and unlikely to happen due to nature of language.
- No decent GUI built-in to R. Tk is available within base R, and packages for other graphical tooklits (e.g. Gtk2, Qt) are also available.

Brief comparison to matlab

- Flexible language, similar to matlab, but definitely not "everything is a matrix". Frames, lists, vectors ...
- From matlab to R: `http://cran.r-project.org/doc/contrib/R-and-octave.txt`
- Comprehensive matlab and R guide: `http://www.math.umaine.edu/faculty/hiebeler/comp/matlabR.html`
- Use x[i] not x(i) for indexing vectors.
- Making vectors: x <−c(10, 9, 5, 1)
- Assignment: best to use <− rather than =. Stay away from underscore!

```
x <- 10
x = 10   ## equivalent, more readable?
lo.val <- 100  ## not lo_val <- 100
```


Using R

- Start-up: type 'R' at command line.
- Type commands interactively, and get results.
- Type commands into a file; source('myfile.R'); edit file ...
- Mac/Win has a GUI for interactive use, with internal editors.
- All platforms have a command-line interface
- Many external editors have support for R, including Emacs (`http://ess.r-project.org`) and Eclipse IDE (`http://www.walware.de/goto/statet`).

My very first R session

```
x <- rnorm(50, mean=4)
x
mean(x)
range(x)
hist(x)
## check help -- how to change title?
?hist
hist(x, main="my first plot")
q()
```


Interacting with R

- Can use up/down arrow keys to go through command history. Within a command, use left/right arrow keys to edit.
- History can be saved over sessions (?history).
- Multiple commands can be put onto one line, using ";" as separator between lines, e.g. x<−10; y<−3; a <−5.
- TAB can do object/file completion.

Objects and Functions

R manipulates objects. Each object has a name and a type (vector, matrix, list, ...)
Name of an object: letters (upper/lower case are distinct), digits, period. Start with a letter.
Objects set by way of assignement. Use the assignment operator rather than = wherever possible. (Does "i = i+1" make sense?)

```
x <- 200
half.x <- x/2
threshold <- 95.0
age <- c(15, 19, 30)
age[2]       ## [] for accessing element.
length(age) ## () for calling function.
```


What's up with the assignment and underscore? (Advanced)

Historically, underscore was used in S for assignment (because an old system keyboard had a key equivalent to the ASCII underscore that generated a back arrow). Hence underscore was not used within variables.
More recently, = is now available as an assignment operator (similar to languages like C), but is frowned upon as it can be confusing.
What does i = i+1 imply mathematically?
Better to stick to `i <-i + 1` and use equals just within calls to functions, e.g. `runif(max=3)`.
Note also that assignments return values:

```
y <- 1 + (x<-9)
a <- b <- 0
```

http://developer.r-project.org/equalAssign.html

Outline

Vectors
Calling functions
Scripts
Matrices
Boolean logic
Lists
Factors
Character arrays
Objects in your environment
Basic plotting
Reading/writing data to file system
Writing functions
Conditionals and looping
Vectorization
Random number generation
Debugging

Vectors

Vectors are a fundamental object for R. Scalars are treated as vector of length 1.

```
y <- c(10, 20, 40)
y[2]
length(y)
x <- 5
length(x)
```

Some operations work element by element, others on the whole vector, compare:

```
y <- c(20, 49, 16, 60, 100)
min(y)
range(y)
sqrt(y)
log(y)
```

Generating vectors

Many short hand methods for regular sequences; c() for irregular.

```
x <- seq(from=1, to=9, by=2)
y <- seq(from=2, by=7, length=3)
z <- 4:8
a <- seq.int(5)                    ## fast for integers
b <- c(3, 9, 2)
d <- c(a, 10, b)
e <- rep( c(1,2), 3)
f <- integer(7)
```


Accessing and setting elements

```
x <- seq(from=100, by=1, length=20)
x[3]          ## just element 3.
x[c(12,14)]   ## element 12 and 14
x[1:5]
bad <- 1:4
x[-bad]       ## exclude elements
```

Can also provide a logical vector of same length as vector (logical values explained later).

```
x <- c(5, 2, 9, 4)
v <- c(T, F, F, T)
x[v]
```

Elements can be set in several ways

```
x <- rep(0,10)
x[1:3] <- 2
x[5:6] <- c(-5, NA)
x[7:10] <- c(1,9)                 ## recycling.
```


Recycling rule (Advanced)

Recycling is convenient, but dangerous; when vectors are of different lengths, the shorter one is often recycled to make a vector of the same length.

```
a <- c(1,5) + 2
x <- c(1,2); y <- c(5,3,9,2)
x + y
x + c(y,1)   ## odd recycling, warning.
```


Naming indexes of a vector

```
joe <- c(24, 1.70)
joe
names(joe)
names(joe) <- c('age', 'height')
joe
joe["height"] == joe[2]
```

Refering to index by name rather than by position can make code more readable, and flexible. Cannot do things like x[1:4] easily though, since you need to name all four elements you want.

Note: in second use of names() above, we are actually using the *replacement function* names<-, see later.

Common functions for vectors

- length()
- rev()
- sum(), cumsum(), prod(), cumprod()
- mean(), sd(), var(), median()
- min(), max(), range(), summary()
- exp(), log(), sin(), cos(), tan() [radians, not degrees]
- round(), ceil(), floor(), signif()
- sort(), order(), rank()
- which(), which.max()
- any(), all()

Functions can be called within function calls; the following are equivalent:

```
x <- c(3, 2, 9, 4)

y <- exp(x); z1 <- which(y > 20) ## case 1
z2 <- which ( exp(x) > 20)       ## case 2

all.equal(z1, z2)
```


Outline

Vectors
Calling functions
Scripts
Matrices
Boolean logic
Lists
Factors
Character arrays
Objects in your environment
Basic plotting
Reading/writing data to file system
Writing functions
Conditionals and looping
Vectorization
Random number generation
Debugging

Default values for function arguments

A function will error if not all required arguments are provided. Some functions have both required and optional arguments. If the optional arguments are not provided, they are either ignored, or they take a default value.

```
Usage:
     round(x, digits = 0)
```

```
x <- c(2.091, 4.126, 7.925)
round()       ## required arg is missing
round(x)
round(x, digits = 2)
```

Let's see how this works in mode detail.

Argument matching

R has a flexible method for specifying arguments to function. We can either provide an actual value for a formal argument, or give arguments as key=value (or formal=actual). As an example, let's look at help for seq:

```
seq(from = 1, to = 1, by = ((to - from)/(length.out - 1)),
    length.out = NULL, along.with = NULL, ...)
```

Typical calls are as follows:

```
seq(1, 3, 0.5)            ## positional matching
seq(1, 5,length.out=3)    ## can skip args (e.g. by)
seq(to=5)                 ## order not important.
seq(f=5,t=1)              ## abbrev tags.
seq(len=5, 1,2)           ## tags removed before positional
```

The ... notation will allows for other arguments to be passed, which are not used by this function.
(NB: in seq(from=x), from is the **formal argument** of the function, and here x is the actual value.)

. . . in function calls (Advanced)

Why do some functions, like sqrt, require only one argument, yet others take many arguments?
Functions like c, cbind, have ... in the arguments:

```
Usage:

     c(..., recursive=FALSE)

Arguments:

     ...: objects to be concatenated.
```

The ... indicate any number of objects may be passed, not just (say) one or two.
The result of c() is to combine them all into one long vector, taking into account if the keyword "recursive" is provided [when args are first flattened].
The ... can also indicate that other arguments can be provided which are not processed directly by this function, but may be useful for other functions (e.g. popular when plotting).

Replacement functions (Advanced)

```
x <- 1:5
x
length(x)
length(x) <- 2
x
```

normally length(x) would return a value, rather than you assigning a value to the function!. These are **replacement functions**, see help page:

```
Usage:

     length(x)
     length(x) <- value
```


Getting help: key commands

- help(hist) to see help file (or ? hist).
- args(hist) to see arguments of a function.
- example(boxplot) run examples in help page.
- help . start () starts web-browser for help/ on-line docs.
- help . search ("histogram")
- demo() to list all demos, e.g. demo(graphics)

NB: ?command works as shorthand for help("command") except for a small number of commands, e.g. if, while. Use the longhand for these.

Help pages

- What you can expect to find:
  - Description – one line summary
  - Usage – formal arguments
  - Arguments – interpretation of arguments
  - Details – what the function does
  - Value – return value.
  - References – documentation
  - See also – helps you find related pages
  - Examples – guaranteed to run: example(hist)

Numbers and special values

- numeric (floating-point, double): 12, 4.92, 1.5e3 is.numeric() (integers converted to f.p.)
- complex: 3+2i. is.complex()

Special values:

- NA: not available. (Often used to represent missing data point) is.na()
- NaN: not a number. e.g. 0/0. is.nan()
- Inf, -Inf: $\pm\infty$ is.finite()

You might also meet:

- NULL: often, list of zero length. is.null()

Operator precedence ?Syntax

```
3 * 4 + 2 != 3 * (4 + 2)
2^3+1   != 2^(3+1)
1:5-1 ## caught me out.
```

Subset taken from ?Syntax, see that page for full list. Highest precedence at top.

```
'[ [['                  indexing
'$ @'                   component / slot extraction
'^'                     exponentiation (right to left)
'- +'                   unary minus and plus
':'                     sequence operator
'%any%'                 special operators
'* /'                   multiply, divide
'+ -'                   (binary) add, subtract
'< > <= >= == !='       ordering and comparison
'!'                     negation
'&  &&'                 and
'| ||'                  or
'<- <<-'                assignment (right to left)
'?'                     help (unary and binary)
```

Bottom line: use parentheses (parens) to order preference.

Operators

Most operators will be familiar, but some may not:

```
x <- 10
x == 4       ## test for equality
x != 10      ## not equal?
7 %/% 2      ## division, ignoring remainder. (3)
7 %% 2       ## remainder (1)

x <- 9       ## assignment
x <<- 9      ## assign x to 9 in the global env. (BAD)

## Raising to a power can be done in two ways.
all.equal( 10.1 ** 2.5, 10.1^2.5)
```


When things go wrong

Syntax errors are those where you've just made a typing mistake.
Logical errors are harder to find!

Common problems:

- missing close bracket leads to continuation line.

  ```
  > x <- (1 + (2 * 3)
  +
  ```

  Hit Ctrl C (below) or keep typing!
- too many parens: 2 + (2*3))
- wrong/mismatched brackets (see next slide).
- Likewise, do not mix double quotes and single quotes.
- ...
- wrong variable name (not syntax error)
- When things seem to take too long, try C-c [Ctrl and C, together]

Types of parentheses

- f(3,4) – call the function f, with arg1=3, arg2=4.
- a + (b*c) – use to enforce order over which statements are executed.
- { expr1; expr2; ... exprn } – group a set of expressions into one compound expression. Value returned is value of last expression; used in looping/conditionals.
- x[4] – get the 4th element of the vector x.
- l[[3]] – get the 3rd element of some list l, and return it. (compare with l[3] which returns a list with just the 3rd element inside).

From interactive to source files

- Typing in commands interactively is good for one-liners, but soon you will want to switch to putting your sequence of commands into a script file, and then ask R to run ('source') those commands.
- This leaves to a rapid edit–run–edit cycle.
- e.g. type these commands into a file:

```
## Example script file, to generate a little plot.
## Demonstrate trigonometric functions.
## Sept 2007
x <- seq(from=0, to=2*pi, length=100)
y <- sin(x)
z <- cos(2*x)
z                                      ## will not appear
print(y)                               ## should use print()
plot(x, y, type='l')
lines(x, z, type='l', col='red')
```

- Eval within R using source('trig.R').

Outline

Vectors
Calling functions
Scripts
Matrices
Boolean logic
Lists
Factors
Character arrays
Objects in your environment
Basic plotting
Reading/writing data to file system
Writing functions
Conditionals and looping
Vectorization
Random number generation
Debugging

Scripts

- Use source('trig.R', echo=T) to see commands and output. Or use print(x) to print an object within a script.
- Keep your code open in the editor in one window, and keep R running in another window.
- Are you in the right directory? Check that you can see your script file in the same directory as where R is currently. Check dir(), and setwd, see later.
- On unix, the initial directory is the directory from where you started R. On windows, the initial directory might be "My Documents". You may need to change directory (setwd) first.
- Use a good editor that helps you spot mistakes (e.g. paren matching). Examples: Emacs/ESS (disclaimer!), gedit.
- Use ".R" or ".r" as the filename suffix. Avoid any temptation to put spaces (although R does not mind) in your filenames!

Why are scripts a good thing?

- You don't have to remember what commands you ran, they are saved in the file.
- This corresponds to the "source is real" philosophy of using S/R.
- You can easily give your work to others, by passing them the file.
- You can eventually run your scripts in BATCH, i.e. non-interactively. Good for long jobs which you can leave overnight.

Running scripts in batch (Advanced)

- At the command line, type "R CMD BATCH trig.R". R will start up, process your commands and then quit.
- Output is stored in the file trig.Rout
- If there were no errors, the last line of the output file shows the time taken to run the script.
- Any output is not shown on the screen but sent to a PDF called `Rplots.pdf`.
- This is a GREAT way of testing your scripts, since R starts with an empty workspace, you will see if you have all the steps needed.
- Aim to always leave your scripts in a working state at the end of a session, so that a few days later you don't have to remember why it wasn't working!

Commenting your work

- Do not be shy when putting comments into your code.
- Meaningful variable names help, but do document. At a bare minimum, each file should state at the top what the purpose of the file. Important variables and functions should be clearly documented.
- You may think it obvious how your code works, but try looking at it a week or a month later and then see if you clearly understand it. If in doubt, document it.
- Describe *what* your code is doing, not *how* it is doing it (WSS, p79). Compare the following two:

```
s <- s + 1    ## prepare to process next subject
j <- j + 1    ## increment j by 1.
```

- Comments can be put before commands, if you temporarily do not want to run that command; remove the comments when you want to run the command again, or delete the line.

```
## x <- c(x, c(1,2,3))
```


Line wrapping

- Line-wrapping. Do not write beyond around column 72, for readability. You can break long expressions at suitable points.
- End of line shold not look like end of an expression. Compare:

```
## 1: ok - all fits onto one line, just.
x <- sqrt( c(100, 200, 300, 400, 500) ) + 10

## 2: not okay -- first line is seen as complete.
x <- sqrt( c(100, 200, 300, 400, 500) )
+ 10

## 3: solved, by moving the operator (+) up.
x <- sqrt( c(100, 200, 300, 400, 500) ) +
10

## 4: as 3, but indentation makes it clearer.
x <- sqrt( c(100, 200, 300, 400, 500) ) +
       10
```

Outline

Vectors
Calling functions
Scripts
Matrices
Boolean logic
Lists
Factors
Character arrays
Objects in your environment
Basic plotting
Reading/writing data to file system
Writing functions
Conditionals and looping
Vectorization
Random number generation
Debugging

Matrices

A matrix is just a vector with some additional markup to reformat it. Matrix stored in column-major order (like fortran, unlike C).

```
x <- 1:6
is.matrix(x)
dim(x) <- c(2,3)
is.matrix(x)
x
dim(x)
x[2,2]
x[1,]                          ## extracting values.
x[1:2, 2:3]
x[,2]                          ## not column vector!
x[,2,drop=F]                   ## gotcha!
```

```
> x
     [,1] [,2] [,3]
[1,]    1    3    5
[2,]    2    4    6
```


Typical matrix construction methods

- matrix()
- cbind()
- rbind()

```
m <- matrix( floor(runif(6, max=50)), nrow=3) ##ncol=2
x <- rbind( c(1,4,9), c(2,6,8), c(3,2,1))
y <- cbind( c(1,2,3), 5, c(4,5,6))  # recycling again
```

Note that matrix indices can also be named:

```
dimnames(m) <- list(student=c("ann", "bob", "joe"),
                    exam=c("math", "french"))
m["bob",]  ## get bob's scores
```


Common matrix operations

- diagonal: diag(x) ## watch if x matrix or scalar.
- matrix multiplication: %*% vs * (element-wise)

  ```
  x <- matrix(1:4, 2,2)
  i <- diag(2) ## 2x2 identity matrix
  x %*% i      ## should be x
  x  *  i      ## not x!
  ```

- transpose: t(x)
- dim, nrow, ncol
- inverse: solve(x), x %*%solve(x) == diag(nrow(x))
- Arrays as extension of matrices to multiple dimensions. x <-array(1:12, c(2,2,3)).

Outline

Vectors
Calling functions
Scripts
Matrices
Boolean logic
Lists
Factors
Character arrays
Objects in your environment
Basic plotting
Reading/writing data to file system
Writing functions
Conditionals and looping
Vectorization
Random number generation
Debugging

Boolean values ?logical

Logical values TRUE/FALSE (abbrev to T/F).

TRUE/FALSE equivalent to 1/0; as.integer(TRUE) is 1.

```
d <- c(3.2, 1.0, 4.0, 9.2, 2.3, 8.1, 6.3)
d > 5.0
d[d> 5.0]
which(d>5.0)
d[ which(d>5.0)]
medium.sized <- (d > 3.0) & (d< 5.0)
d[medium.sized]
d[-medium.sized]
ifelse(d > 3.0, 1.0, 0.0)      ## Very handy!
```

Key operators for handling boolean values:

```
!TRUE                  ## negation: swap T -- F.
TRUE  & FALSE          ## and: both must be true.
FALSE | TRUE           ## or: one must be true.
xor(TRUE, TRUE)        ## xor: only one is true.
```


Boolean logic: issues

a & b (same for a | b) is an elementwise operation, with a result the same length as the longer of a, b (recycling is used if one vector is shorter).

a && b examines only the first element of a and b, returning one logical value. **Lazy evaluation** is used: we calculate only what's needed to determine result.

```
TRUE || some.long.computation()
TRUE && stop("no")
```

Comparing numbers: When testing numbers for equality, can use x == y when x,y are integers, otherwise use all.equal(x,y). See later on numerics. Avoid using F where possible: F <-3; F == FALSE

Outline

Vectors
Calling functions
Scripts
Matrices
Boolean logic
Lists
Factors
Character arrays
Objects in your environment
Basic plotting
Reading/writing data to file system
Writing functions
Conditionals and looping
Vectorization
Random number generation
Debugging

What is a list?

A list is used to collect a group of objects of different sizes and types. Very flexible. Often returned as the result of a complex function (e.g. model fit) to return all relevant information in one object.

```
l <- list(id='joe', height=1.70, dob=c(1960, 12, 1))
l
length(l)
names(l)                        ##show components
l$height                        ##access an element.
unlist(l) ## compact way of viewing it.
```

List elements can either be accessed by name (e.g. l$height) or by position (l [[2]]).
When using numbers to index list, compare l[2] (a list with one element) with l[[2]]. You can therefore do l[2:3] but not l[[2:3]].

Modifying lists (Advanced)

We can append new items to list either by making a new list from the old one (e.g. 1) , or directly by assigning new element (e.g. 2):

```
l1 <- list(who="fred")
l1 <- c(l1, height=1.8)          ## e.g. 1
l1[["dob"]] <- c(1965, 10, 17) ## e.g. 2
```

Deleting list items:

```
l1["height"] <- NULL
```

Finally, for completeness, here is a way to predefine a list of given length and gradually fill it in:

```
empty <- vector("list", 3) ## Prealloc to given length.
names(empty) <- c("who", "height", "dob")
empty[["height"]] <- 1.8
```


Data frames

Data frame is a special kind of list; all elements are vectors of same length.
This is like a matrix, but each column can be of a different type.
Useful for reading in tabular data from a file (see read.csv).

```
names <- c("joe", "fred", "harry")
a <- c(24, 19, 30)
ht <- c(1.7, 1.8, 1.75)
s <- c(TRUE, FALSE, TRUE)
d <- data.frame(name=names, age=a,
                height=ht, student=s)
d$age
names(d)
d[2,] ## access 2nd row.
```

Compare how a data frame (d) is printed, compared to printing as.list(d)

Outline

Vectors
Calling functions
Scripts
Matrices
Boolean logic
Lists
Factors
Character arrays
Objects in your environment
Basic plotting
Reading/writing data to file system
Writing functions
Conditionals and looping
Vectorization
Random number generation
Debugging

Factors (Advanced)

(Mostly seen when reading in data from e.g. CSV file)
Factors internally code categorical variables with a number. e.g. 1=Sunday, 2=Monday, ... 7=Saturday. For large vectors, this is more efficient storage, especially when character strings repeat. Can also make code more readable.

```
scores1 <- c('good', 'poor', 'bad', 'poor',
             'bad', 'bad', 'good')
scores <- factor(scores1)
scores
levels(scores)
as.integer(scores)
which(scores1 == 'bad')

## Can do further comparisons with an ordered factor
## Levels are now ordered, as shown by "<" in levels.
s2 <- factor(scores1, levels=c('poor', 'bad',
             'good'), ordered=T)
s2[1] > s2[2]
```


Outline

Vectors
Calling functions
Scripts
Matrices
Boolean logic
Lists
Factors
Character arrays
Objects in your environment
Basic plotting
Reading/writing data to file system
Writing functions
Conditionals and looping
Vectorization
Random number generation
Debugging

Strings / character arrays

Character arrays are vectors of strings.

- Use single (') or double (") quotes to mark strings, but don't mix:

```
x <- 'good'
z <- "no'
z <- "it's working"
```

- Within a script, easy way to generate output:

```
cat("Now computing the steady-state\n")
x <- 134
cat("sqrt of", x, "is", sqrt(x), "\n")
cat("sqrt of", x, "is", sqrt(x), "\n", sep='__')
```

- blackslash characters allow you to generate control characters, importantly: newline: \n, tab: \t. e.g. cat("5\t9\n")
- paste() returns string, e.g. for assignment.

```
x <- 1:5; exp.dir <- '/home/stephen/res'
file <- paste(exp.dir, '/expt_res', x, '.dat', sep='')
```


Strings

- Just as R stores vectors of numbers, it also stores vectors of strings.
- Pattern matching facilities are available, based on Unix terms (grep, regular expressions). These are worth learning:

```
s <- c('apple', 'bee', 'cars', 'danish', 'egg')
nchar(s)
substr(s, 2,3)
grep('e', s)
grep('^e', s)   ## regexps...
 sub('e', '_', s)
gsub('e', '_', s)  ## global sub, watch "bee"

toupper(s)
sprintf('name %s len %d', s, nchar(s)) ## C users!
```

Outline

Vectors
Calling functions
Scripts
Matrices
Boolean logic
Lists
Factors
Character arrays
Objects in your environment
Basic plotting
Reading/writing data to file system
Writing functions
Conditionals and looping
Vectorization
Random number generation
Debugging

Inspecting variables and the environment

is.xyz, as.xyz family of functions are useful for checking mode of objects and converting between them. (e.g. is.vector).

```
objects()              ## what vars do I have?
ls()                   ## shorthand for objects.
rm(list = ls()) ## clear up the working environment
x <- 9; y<- c(2,4,5); m <- matrix(2:5, 2,2)
ls()
rm(x)           ## remove a var
mode(y)
object.size(y)
is.vector(y)
is.matrix(y)
as.vector(m) ## convert from one family to another.
```


What is an object?

- An object is typically either a variable or a function.
- You can use the same name for a function and a variable, and R uses context to decide which you mean:

```
> sum <- 3 + 4 + 5
> total <- sum(1:4)
> total
[1] 10
> sum
[1] 12
> sum(sum)  ## can get confusing!
[1] 12
```


Outline

Vectors
Calling functions
Scripts
Matrices
Boolean logic
Lists
Factors
Character arrays
Objects in your environment
Basic plotting
Reading/writing data to file system
Writing functions
Conditionals and looping
Vectorization
Random number generation
Debugging

Basic plotting

- Basic x,y plots
- Multiple plots in one figure
- Saving your plots

This section will just introduce the mechanics of making basic plots, rather than worry about interpreting them.

Basic plotting

```
x <- seq(from=0, to=2*pi, len=1000)
y <- cos(2*x)
## just provide data; sensible labelling
plot(x,y)

## Expand on previous plot ...
plot(x,y, main='cos(2x)', type='l', lty=1, bty='n')
y2 <- sin(2*x)
lines(x,y2, main='sin(2x)', type='l', lty=2)
same <- which( abs(y - y2) < 0.01)
points(x[same], y[same], pch=19, col='red', cex=3)
legend('bottomright', c("cos(2x)", "sin(2x)"),
       lty=c(1,2))
```


Options controlling the plot

par() outputs the (long) list of options that control plotting behaviour.
Read ?par for all the details!
Common options to explore:

- mfrow, mfcol: multiple plots in figure
- mar, oma: margins around plot and figure.
- ask: whether to hit RETURN between pages of figures.

Mutltiple data sources on one plot

When you wish to have multiple data sources on one plot (e.g. two time-series plots), the approach is to draw the first using plot and then draw subsequent features using lines or points.
Axes are not rescaled, so draw the bigger plot first.

```
x <- 1:30
y <- sqrt(x); z <- log(x)
plot(x,y); lines(x,z, col='red')
plot(x,z); lines(x,y, col='red') ## some data missing
```

Multiple plots in one figure

mfrow and mfcol are useful parameters within par(), but margins often need to be changed to maximise space.

```
pdf(file='mfrow_eg.pdf', width=6,
    height=4)
par(mfrow=c(2,3))
par(mar=c(3.5, 3.5, 1.5, 0.5),
    mgp=c(2.5, 1, 0))
x <- seq(from=0, to=2*pi, len=100)
plot(x, sin(x), main="sin (x)",
     type='l')
plot(x, sin(2*x), main="sin (2x)",
     type='l')
plot(x, sin(3*x), main="sin (3x)",
     type='l')
plot(x, cos(x), main="cos (x)",
     type='l')
plot(x, cos(2*x), main="cos (2x)",
     type='l')
plot(x, cos(3*x), main="cos (3x)",
     type='l')
dev.off()
```

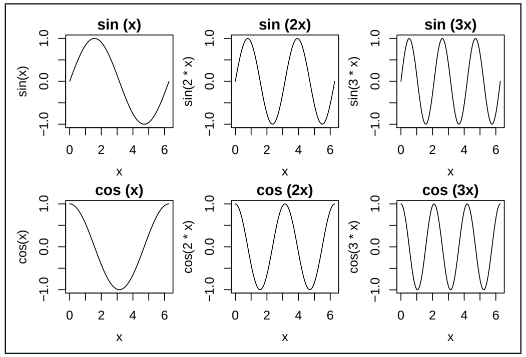

Saving your plots

R can save plots in many formats, including PDF, postscript, PNG, JPEG. Best to use vector formats (PDF, postscript) for graphs and bitmap formats (png, jpeg) for images.
R has output devices, only one of which is active, dev.cur().

```
dev.list()
pdf(file='hist.pdf', width=7, height=7) ## inch
dev.list()
hist( rnorm(9999) )
dev.off()                          ## close device

png(file='hist.png', w=600, h=600) ## pixels
hist( rnorm(9999) )
dev.off()
```

Zoom in on text of PNG to see limitations of this format.

Next steps with plotting (Advanced)

R has a vast range of functions for plotting particular data types. You may read about different packages for plotting:

- base graphics (or "traditional")
- lattice/grid (lattice is built upon grid)
- ggplot (quite new)
  `http://had.co.nz/ggplot2/`

Here are some starting points to explore:

- demo(graphics) to see diversity of plots.
- low-level functions: symbols(), rect(), segments(), abline().
- R graphics gallery
  `http://addictedtor.free.fr/graphiques`

Outline

Vectors
Calling functions
Scripts
Matrices
Boolean logic
Lists
Factors
Character arrays
Objects in your environment
Basic plotting
Reading/writing data to file system
Writing functions
Conditionals and looping
Vectorization
Random number generation
Debugging

Reading/writing data to file system

- What's my current directory? dir , getwd, setwd
- scan, readLines
- read.csv, read.table, write.table
- RData files
- Further I/O functions

Interacting with the file system

- where am I currently? getwd()
- change me to a new directory: setwd("/tmp")
  (GUIs have chooser for interactively changing directory.)
- What files are in my [current] directory?

```
dir()
dir("/tmp")
dir(pattern="\\.R$")  ## regexps, see later.
```


Scan, write, readLines

For basic reading/writing of data, use scan/write. Filenames are specified relative to current directory. Can even give URL as a file. Files often have a header which can be skipped over.

```
x <- scan('ages.dat', skip=1)
summary(x)

## No line break in next line...
h <- scan('http://www.damtp.cam.ac.uk/
user/eglen/teaching/r/heights.dat}')

rand.vals <- round( runif(100, min=5, max=10), 2)
write(rand.vals, '/tmp/randvals.dat')
s <- scan('/tmp/randvals.dat')
all.equal(s, rand.vals)

s1 <- readLines('ages.dat')  ## treats as strings
```


read.table / read.csv / write.csv

If data are tabular, read.table or read.csv is often useful. (Useful for importing spreadsheets; just save as a comma separated value file, CSV.)

```
x <- read.table('../data/players.dat',
                sep='\t',header=T)
names(x)
x
x[2,]
x$age
is.data.frame(x)
write.csv(x, '/tmp/players.csv', row.names=F)

## sort by goals scored.
x[order(-x$goals),]
```

See ?read.table.

Rdata files

Text files are useful for portably storing data, so that they can be read across applications. R has its own format for *efficiently* storing objects. Files much smaller than text files. However, this format is not universally known.

```
n <- 99999; x <- rnorm(n)
txt.file <- '/tmp/my_rnorm.txt'
rda.file <- '/tmp/my_rnorm.rda'
write(x, n, file=txt.file)
save(x, n,  file=rda.file)

## Compare sizes of files with the object.
object.size(x)
file.info(txt.file)$size
file.info(rda.file)$size   ## compression?

rm(x,n)
load(rda.file) ## reload data.
```


Saving your workspace with .RData files

When you quit R, you are asked:

```
> q()
Save workspace image? [y/n/c]:
```

If you answer y, all objects in your global environment are saved for future use, using save.image. From ?save:

```
'save.image()' is just a short-cut for "save my current
workspace", i.e., 'save(list = ls(all=TRUE), file = ".RData")'.
It is also what happens with 'q("yes")'.
```

If an `.RData` file is present in your current directory when you start R, it is silently loaded. This may be useful, but I think it can be dangerous, as you may not realise what values have been silently loaded.
Here, "all objects" means all your variables and functions.

Further I/O functions (Advanced)

R has many facilities for I/O. See for example the following help topics.

- ?connections — interface to files, pipes, sockets, compressed files . . .
- ?sink — divert R output to a connectin
- ?dget / ?dput — read/write ASCII representation of an R object.

Outline

Vectors
Calling functions
Scripts
Matrices
Boolean logic
Lists
Factors
Character arrays
Objects in your environment
Basic plotting
Reading/writing data to file system
Writing functions
Conditionals and looping
Vectorization
Random number generation
Debugging

Writing functions: overview

- Why bother?
- How to write (local args, return value; cannot change value)
- Example: computing std. deviation
- Local variables within functions
- Recursion.

Functions

- Functions promote code reuse.
- Black-box approach; given inputs, what output should I expect? This requires good documentation of what your function does. Can it be described without having to look at the code?
- Finding the right level of definition for a function is hard, and how to modularise comes with experience. Typically rewrite many times before getting final solution
- How to define a new function:

```
my.fun <- function(arg1, arg2, ...) {
  ## Doc string here.
  x <- arg1 * 2
  y <- sqrt(arg2) + 5
  z <- x * y
  ## last value is the return value of the function.
  ## Use a list to return several items.
  z
}
```


Example of writing a new function

Compute the standard deviation of a vector of numbers:

$$std.dev = \sqrt{\frac{\sum_{i=1}^{n}(x_i - \bar{x})^2}{n-1}} \quad \text{where} \quad \bar{x} = \frac{\sum_{i=1}^{n} x_i}{n}$$

```
std.dev <- function(x) {
  ## Return std dev of X.
  n <- length(x)
  xbar <- sum(x)/n
  diff <- x - xbar
  sum.sq <- sum( diff^2)
  var <- sum.sq / (n-1)

  ## last value calculated is return value.
  sqrt(var)
}
```


Terminology of variables within functions

- In std.dev, x is the name of a **formal argument**. In the following, y is called the **actual argument** (doesn't have to be named x – can be named however you wish).

```
n <- 5
y <- c(9, 2, 7, 10)
std.dev(y)
print(n) ## should still be 5, not 4.
```

- **Local variables** within function are not available outside of function.
- Any change to formal args within a function does not change value of actual argument outside the function:

```
sum.sq <- function(x) {
  x <- x^2   ## change internally
  sum(x)
}
y <- c(4, 5, 6)
sum.sq(y)
y
```

Handling unbound variables

Variables created by assignment within a function are known as local variables (e.g. y below). If a variable is not a local variable, or formal argument, then we call it an **unbound variable**. An unbound variable may then be found in the enclosing environment (typically the global workspace), or if it cannot be found, an error is generated.

```
fn1 <- function(x) {
  y <- x^2
  res <- sum( (y - thresh)^2 )
  res
}

dat <- 1:5
fn1(dat)     ## case 1

thresh <- 10
fn1(dat)     ## case 2
```

Advanced: use codetools :: checkUsage() to find unbound vars.

Handling unbound variables (2)

In this case, better to define thresh as an argument of the function, and provide a default value:

```
fn1 <- function(x, thresh=10) {
  y <- x^2
  res <- sum( (y - thresh)^2 )
  res
}
fn1(dat)     ## case 3
```


Writing a replacement function (Advanced)

Convention for a replacement function is that the name should end with <-. The last argument of the replacement function must be called VALUE and is the RHS of the assignment.

```
"threshold<-" <- function(x, value) {
  ## X is the object to update
  ##  VALUE is the value on the RHS.
  y <- ifelse(x>value, 1, 0)
  y                             ## return new value.
}
x <- c(0.3, 0.1, 0.6, 0.7, 0.9, 0.2)
threshold(x) <- 0.4
x
```


Tips for writing functions

- Can you think of a way to break down the problem so that a team can work on the problem, with each person assigned to a independent piece? "Divide + conquer".
- Each function should be easy to test, then you can "freeze" it. Write test cases, which can be automatically checked.

  ```
  all.equal(my.fun(100,200), 300)
  ```

- Rule of thumb: each function should be no more than a page or two of code.
- For large projects, avoid mixing computation and plotting in the same function – separate the two jobs; this makes it easier to run in batch.

  ```
  res <- some.computation(par1, par2, par3)
  plot.results(res)
  ```

Outline

Vectors
Calling functions
Scripts
Matrices
Boolean logic
Lists
Factors
Character arrays
Objects in your environment
Basic plotting
Reading/writing data to file system
Writing functions
Conditionals and looping
Vectorization
Random number generation
Debugging

Control-flow constructs

- if
- switch
- for
- while
- Vectorization
- simple applications – numerics

if / if ... else ...

```
x <- 8;

if (x > 10) {
  ## condition was true
  cat("x is bigger than 10\n")
} else {
  cat("x is 10 or less\n")
}
```

Notes:
"else ..." can be omitted if you do not need it.
if returns a value, which can be assigned, e.g. y <- if (x <10) 40 else 20.
A better solution in this case however is the vectorized form
y <- ifelse (x<10, 40, 20)

Braces in conditional constructs

Curly braces not needed if there is only one expression in the if clause:

```
if ( x > 10 ) {
  y <- 1
}
```

```
if ( x > 10 )
  y <- 1
```

But braces are needed in multiline if/else statement:

```
if ( x > 10 ) {
  y <- 1
} else {
  y <- 0   ## OK
}
```

```
if ( x > 10 )
  y <- 1
else
  y <- 0   ## NOT OK
```

From ?Control: Note that it is a common mistake to forget to put braces ('{ .. }') around your statements, e.g., after 'if(..)' or 'for(....)'. In particular, you should not have a newline between '}' and 'else' to avoid a syntax error in entering a 'if ... else' construct at the keyboard or via 'source'. For that reason, one (somewhat extreme) attitude of defensive programming is to always use braces, e.g., for 'if' clauses.

switch (Advanced)

Nested if ... else commands can get a bit messy. Like other languages, R has a switch construct. From ?switch:

```
centre <- function(x, type) {
  switch(type,
         mean = mean(x),
         median = median(x),
         trimmed = mean(x, trim = .1))
}
x <- rcauchy(10)
centre(x, "mean")
centre(x, "median")
centre(x, "trimmed")
```


Recursive functions

Here is an example of using conditionals with a divide and conquer approach; quicksort in a few lines (albeit not very efficient). qsort.R

```
qsort <- function(data) {
  ## Sort DATA into ascending order.
  n <- length(data)
  if (n <= 1) {
    data
  } else {
    pivot   <- data[floor(n/2)]
    less    <- data[which(data <  pivot)]
    equal   <- data[which(data == pivot)]
    greater <- data[which(data >  pivot)]
    c( qsort(less), equal, qsort(greater))
  }
}

replicate(99, {
  data <- runif(2000, max=10)
  all.equal(qsort(data), sort(data)) } )
```


Looping constructs

Looping constructs allow you to repeat calculations as many times as you wish. This is why computers are so useful – it is just as easy (usually) to repeat something 1000 times as 10 times.

e.g. if you want to simulate flipping a (biased) coin 100 times, and counting the number of heads, no problem. If you want to repeat this process 1000 times, no problem. See later.

for loops

for (var in seq) command

SEQ is a vector; VAR is set in turn to each value in the vector, and then command executed. Multiple commands can be given within braces. e.g.

```
x <- 6
for (i in 1:10) {
  res <- x * i
  cat(x, "*", i, "=", res, "\n")
}
```

while loops

```
while (condition) {
  command
  command
}
```

So the commands are executed until the condition is no longer true.
Typically then one of the commands will change the condition.
e.g. print all the Fibonacci numbers (f[i] = f[i-1] + f[i-2]) less than 100.

```
n1 <- 0; n2 <- 1
while (n2 < 100) {
  print(n2)
  old <- n2
  n2 <- n2 + n1
  n1 <- old
}
```


Breaking out of loops

repeat expr will repeatedly execute expr until you break out of the loop.

```
i <- 3
repeat {
  if (i==10) {
    break
  } else {
    cat("i is", i, "\n")
    i<- i+1
  }
}
```

next allows you to skip to next iteration of a loop. Both next and break can be used within other loops (while, for).

```
for (i in 1:10) {
  if ((i %% 2) == 0)
    next
  print(i)
}
```


A word on indentation

Indentation helps you see the flow of the logic, rather than flattened version. (Use tab key to indent). Reformatting tools are available (e.g. within Emacs).

```
## version 1.
i <- 3
repeat {
  if (i==10) {
    break
  } else {
    cat("i is", i, "\n")
    i<- i+1
  }
}
```

```
## version 2.
i <- 3
repeat {
if (i==10) {
break
} else {
cat("i is", i, "\n")
i<- i+1
}
}
```

Indentation helps to show structure, and match braces.

Outline

Vectors
Calling functions
Scripts
Matrices
Boolean logic
Lists
Factors
Character arrays
Objects in your environment
Basic plotting
Reading/writing data to file system
Writing functions
Conditionals and looping
Vectorization
Random number generation
Debugging

Vectorization

When possible, operate on vectors, rather than using for loops.
Rewrite code, but beware sometimes not possible (Fibonacci). e.g. compute difference between times of events, e. Given n events, there will be n-1 inter-event times. interval[i] = e[i+1] - e[i]

```
diff1 <- function(e) {
  n <- length(e)
  interval <- rep(0, n-1) ## good to pre-alloc!
  for (i in 1:(n-1)) {
    interval[i] <- e[i+1] - e[i]
  }
  interval
}
diff2<- function(e) {
  n <- length(e)
  e[-1] - e[-n]
}

e <- c(2, 5, 10.2, 12, 19)
diff1(e)
all.equal(diff1(e), diff2(e))
```

Advantages: shorter, more readable, faster (no loops).

Vectorization example

Q: Flip a biased coin [p=0.6 of heads] 100 times; how many heads do you get? Repeat this for 1000 trials.

```
n <- 100    ## number of coin flips in trial
p <- 0.6    ## prob of getting heads
ntrials <- 1000
```

```
trial1 <- function(n, p.heads) {
  count <- 0
  for (i in 1:n) {
    if (runif(1) < p.heads)
      count <- count +1
  }
  count
}

res <- rep(0, ntrials)
for (j in 1:ntrials) {
  res[j] <- trial1(n, p)
}
hist(res)
```


Vectorization example

Q: Flip a biased coin [p=0.6 of heads] 100 times; how many heads do you get? Repeat this for 1000 trials.

```
n <- 100    ## number of coin flips in trial
p <- 0.6    ## prob of getting heads
ntrials <- 1000
```

```
trial1 <- function(n, p.heads) {
  count <- 0
  for (i in 1:n) {
    if (runif(1) < p.heads)
      count <- count +1
  }
  count
}

res <- rep(0, ntrials)
for (j in 1:ntrials) {
  res[j] <- trial1(n, p)
}
hist(res)
```

```
trial2 <- function(n, p.heads) {
  rand.vals <- runif(n)
  sum( rand.vals < p.heads)
}

res <- replicate(ntrials,
                 trial2(n, p))
hist(res)
```


Vectorization example

Q: Flip a biased coin [p=0.6 of heads] 100 times; how many heads do you get? Repeat this for 1000 trials.

```
n <- 100    ## number of coin flips in trial
p <- 0.6    ## prob of getting heads
ntrials <- 1000
```

```
trial1 <- function(n, p.heads) {
  count <- 0
  for (i in 1:n) {
    if (runif(1) < p.heads)
      count <- count +1
  }
  count
}

res <- rep(0, ntrials)
for (j in 1:ntrials) {
  res[j] <- trial1(n, p)
}
hist(res)
```

```
trial2 <- function(n, p.heads) {
  rand.vals <- runif(n)
  sum( rand.vals < p.heads)
}

res <- replicate(ntrials,
                 trial2(n, p))
hist(res)
```

In this case, hist(rbinom(1000, 100, 0.6)) would also work!

apply family

e.g. how to compute sum of each row of a matrix? sum(A) will normally return the sum of all elements of A.

```
apply(X, MARGIN, FUN, ...)
MARGIN = 1 for row, 2 for cols.
FUN = function to apply
... = extra args to function.
```

```
A <- matrix(1:6, 2,3)
row.means <- apply(A, 1, mean)
col.sums  <- apply(A, 2, sum, na.rm=T)
```

Other functions: lapply (apply to list), sapply (simplify), replicate.

```
lapply(ls(), object.size)
sapply(ls(), object.size)
hist( replicate(200, mean(rnorm(100)) ))
```


Anonymous functions (Advanced)

Sometimes you don't want to pollute name space by defining a new function, so just use an "anonymous function", i.e. a function without a name. Particularly useful e.g. in an apply call.

```
my.mat <- matrix(1:10, ncol=5)
apply(my.mat, 2, function(x) { sum(x^2)+10 } )
```

Since functions are just objects, anonymous functions are just objects without names, similar to 'anonymous numbers' like a+b in an expression a+b+c.

Fibonacci sequence

$$0, 1, 1, 2, 3, 5, 8, 13, 21, \ldots$$

$$f[n] = f[n-1] + f[n-2]$$

How to vectorize?
Exercise: write a function, fibonnaci(n) that returns the nth element of the sequence. Assume that fibonnaci(1) = 0, fibonacci(2) = 1.
Exercise: use fibonacci() to estimate the golden ratio.

Efficiency

Knuth: "premature optimization is evil" quote (WSS book).
Examples adopted from `www.mathworks.com/res/code_segments`
f1 is bad; should pre-allocate vector, rather than rely on R to allocate memory repeatedly (as seen by high 'system' time).

```
f1 <- function() {
  n <- 1e4; decay <- 0.9995

  out <- 1.0
  for (i in 2:n)
    out[i] <- out[i-1] * decay
  out
}
```

```
f2 <- function() {
  n <- 1e4; decay <- 0.99995
  out <- rep(0, n)    ##pre-alloc
  out[1] <- 1.0
  for (i in 2:n)
    out[i] <- out[i-1] * decay
  out
}
```

```
system.time(o1 <- f1())
system.time(o2 <- f2())
```

Numerics issues

Although integer arithmic is reliable, floating-point arithmetic is to be treated with care! (All R's calculations are in what C programmers call "double precision".)

```
1 + 2 == 3
.1 + .2 == .3
```

From FAQ (7.31?)

```
a <- sqrt(2)
a * a == 2
a * a  - 2
```

How can we find out biggest and smallest numbers that can be stored in a double precision number?

How big is infinity?

Use while loop to estimate it:

```
x <- 1
while ( is.finite(x*2) ) {
      x <- x*2
}

> x
[1] 8.988466e+307
> x*2
[1] Inf
> (x*2)/2
[1] Inf

.Machine$double.xmax
[1] 1.797693e+308
```


How small is epsilon?

How big can ϵ be such that $1 + \epsilon = 1$? (Taken from Goldberg (1991) ACM article, p220).

```
eps <- 1
while (eps + 1 > 1) {
  eps <- eps * 0.5
}
> eps
[1] 1.110223e-16
> 1 + eps
[1] 1
> (1 + eps == 1)
[1] TRUE
> 1 + (2*eps)
[1] 1
> (1 + (2*eps) == 1)
[1] FALSE
```


Outline

Vectors
Calling functions
Scripts
Matrices
Boolean logic
Lists
Factors
Character arrays
Objects in your environment
Basic plotting
Reading/writing data to file system
Writing functions
Conditionals and looping
Vectorization
Random number generation
Debugging

Random number generation

Computers usaully generate "pseudo-random numbers". They are generated based on some iterative formula:

$$x_{new} = f(x_{old}) \mod N$$

where modulo operation provides the "remainder" division.
To generate the first random number, you need a **seed**.
Setting the seed allows you to reliably generate the same sequence of numbers, which can [rarely] be useful when debugging programs.
R has many routines for generating random samples from various distributions (See A.L.'s lecture), but for now we will just use runif(), (and maybe rnorm()).
Exercise: write a random number generator. See: "Randu: a bad random number generator". `http://physics.ucsc.edu/~peter/115/randu.pdf`
Exercise: Apply the central limit theorem to generate samples from a normal distribution by adding together samples from a uniform distribution.

Outline

Vectors
Calling functions
Scripts
Matrices
Boolean logic
Lists
Factors
Character arrays
Objects in your environment
Basic plotting
Reading/writing data to file system
Writing functions
Conditionals and looping
Vectorization
Random number generation
Debugging

Debugging (Advanced)

See **An introduction to the Interactive Debugging Tools in R, Roger D Peng** for detailed usage.
`http://www.biostat.jhsph.edu/~rpeng/docs/R-debug-tools.pdf`

- warnings vs errors; converting warnings to errors; stopifnot().
- what to do when I get an error: traceback()
- simple print statements are often useful.
- Use of browser() at key points in code.
- debug(fn), undebug(fn)
- Using recover() rather than browser()

Warnings and errors

- A warning is softer than an error; if a warning is generated your program will still continue, whereas an error will stop the program.

```
log(c(2, 1, 0, -1, 2)); print('end')  # warning
xor( c(TRUE, FALSE)); print ('end')  # error
```

- If you try to isolate warnings, you can change warnings to errors: options(warn=2). See ?options for further details.
- Add warnings and errors to your code using warning(), stop().
- Can add "assertions" into your code to check that certain values hold.

```
stopifnot(x>0)
```

- Other useful safety checks: all(x>0), any(x>0)

Traceback

When your program generates an error, use traceback() to find out where it went wrong:

```
start <- function() { go( sqrt(10)) }
go <- function(x) { inner(x, '-13')}
inner <- function(a, b) {
  c <- sqrt(b)
  a * log(c)
}

> start()
Error in sqrt(b) : Non-numeric argument to
mathematical function
> traceback()
3: inner(x, "-13")
2: go(sqrt(10))
1: start()
```


Single-stepping through your code

Use browser() to single-step through your code. Place it within your function at the point you want to examine (e.g.) local variables.

Can use debug(function.name) to step through entire function. undebug() will remove that debug call.
Within the browser, you can enter expressions as normal, or you can give a few debug commands:

- n: single-step
- c: exit browser and continue
- Q: exit browser and abort, return to top-level.
- where: show stack trace.

Debug on `stddev.R`

Safety-checks: browser

Here's a possible usage of browser() that I have in my code:

```
find.high <- function(x, t) {
  ## Return samples in x bigger than t.
  ## (Better to use x[x>t] in real-life!)
  max.length <- 100  ## should be upper limit...
  results <- rep(0, max.length)
  counter <- 0
  for (i in x) {
    if (i > t) {
      counter <- counter + 1
      if (counter > max.length) {
        browser()
      } else {
        results[counter] <- i
      }
    }
  }
  results[1:counter]
}
x <- rnorm(100)
find.high(x, 0.7)

x <- rnorm(1000)
(1- pnorm(0.7)) * length(x) ## expected.
find.high(x, 0.7)
```


recover

recover() is like browser, except you can choose which level to inspect, rather than the level at which browser was called.
Following allows recover() to be launched when you hit an error:

```
options(error=recover)
```

Here we simply tell R that when an error is generated, we call the function "recover". The default is NULL, in which case stop is called.

From ?options:

> *Note that these need to specified as e.g. 'options=utils::recover' in startup files such as '.Rprofile'.*

Reproducible research: Sweave and vignettes

- Use one file to store code and document. Best shown by way of example... `estimate.Rnw`
- Vignettes often used in Bioconductor to document packages.
- > library (tkWidgets); vExplorer ()
  Interactively explore vignettes.

Packages

- R has a packaging system for external code.
- A **package** is loaded from a **library** using library (pkg.name).
- Beware: don't call a package a library! A library is a group of folders where packages are stored ...

```
library()                    ## view available packages
library(help=cluster)        ## what's in this?
library(cluster)             ## load package
example(pam)                 ## can use pam and friends.
detach("package:cluster")    ## remove pkg.
```


CRAN: Comprehensive R Archive Network

CRAN: Site(s) for downloading R, and also its many contributed *packages*. Mac/Win have a GUI for installing packages, or it can be done on the command line:

```
library(stats)
library(help=stats)
install.packages(c("splancs", "sp"))
$ R CMD INSTALL mypackage.tar.gz  ## from shell
```

If asked to selected a CRAN mirror, in UK use: `http://www.stats.bris.ac.uk/R`.
If installing on a machine where you do not have root access (e.g. PWF linux), you should add the following to your .bashrc file:

```
export R_LIBS=$HOME/NOBACKUP/RLIB
```

(Be careful! Check that you are not overwriting an existing R_LIBS setting.)

Bioconductor

A success story of R. Started 2001 with aims to:

- provide access to stat/graphical methods for analysis of *genomic* data.
- link seamlessly to on-line databases (PubMed/GenBank).
- allow rapid development of extensible software.
- provide training in methods (short courses).
- promote software with high quality docs and reproducible research (vignettes) ...
- Gentleman et al. (2004) Genome Biology 5:R80.
  `http://genomebiology.com/2004/5/10/R80`

Other topics of interest (Advanced)

- Building your own packages. Useful for packaging up your code, data sets and documentation. You may wish to do this for large projects that you wish to share with others. Read *Writing R Extensions* manual and see `package.skeleton` to get started.
- Access to databases. Computational Biology datasets are often quite large, and you might wish to access data via databases. R package DBI provides common interface to SQLite, MySQL, Oracle. See Gentleman (2008), Chapter 8.
