# Supplementary material for: A Quick Guide to Teaching R Programming to Computational Biology Students
Source: PLoS Comput Biol. 2009 Aug 28;5(8):e1000482. doi: 10.1371/journal.pcbi.1000482 (PMC2725315; doi:10.1371/journal.pcbi.1000482)
Supplement: Text S3 — PDF output from the example Sweave document. (0.11 MB PDF) [file pcbi.1000482.s003.pdf]

Example Sweave document: estimating π

Stephen J Eglen

April 28, 2009

1 Introduction

This is an example document created using the Sweave system (`http://www.statistik.lmu.de/~leisch/Sweave/`). Sweave is a tool for combining both LaTeX documentation and R code within the same file. For this document, the master file is `estimate.Rnw`. This is processed by the Sweave system in R, which runs the R code to generate textual/graphical output, and also creates a LaTeX document. The LaTeX document is then typeset to create the pdf document. On unix/macintosh, the following commands should recreate the pdf file:

```
$ R CMD Sweave estimate.Rnw
$ pdflatex estimate.tex
```

2 Task: estimate the value of π

Our task is to estimate the value of π by simulating darts being thrown at a dartboard. Imagine that the person throwing the darts is not very good, and randomly throws each dart so that it falls uniformly within a square of side length $2r$, with the dartboard of radius r centred within that square. If the player throws n darts, and d of them hit the dartboard, then for large enough n, the ratio d/n should approximate the ratio of the area of the dartboard to the enclosing square, $\pi r^2/4r^2 \equiv \pi/4$. From this, we can estimate $\pi \approx 4d/n$.

We start with an example, using R to draw both the dartboard and the surrounding square, together with $n = 50$ darts. The radius of the dartboard here is 1 unit, although the value is not important.

```
> r <- 1
> n <- 50
> par(las=1)
> plot(NA, xlim=c(-r,r), ylim=c(-r,r), asp=1, bty='n',
+      xaxt='n', yaxt='n', xlab='', ylab='')
> axis(1, at=c(-r,0,r)); axis(2, at=c(-r,0,r))
> symbols(x=0, y=0, circles=r, inch=F, add=T)
> x <- runif(n, -r, r);  y <- runif(n, -r, r)
> inside <- x^2 + y^2 < r^2
> d <- length(which(inside))
> points(x, y, pch=ifelse(inside, 19, 4))
> rect(-r, -r, r, r, border='blue', lwd=2)
```

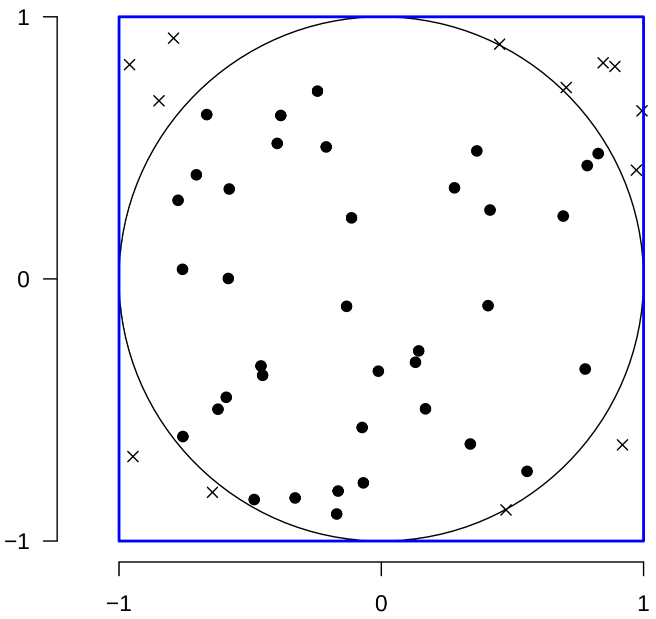

A dart is drawn as a filled circle if it falls within the dartboard, else it is drawn as a cross. In this case the number of darts within the circle is 37, and so the estimated value is $\pi \approx 2.96$.

The estimate of π should improve as we increase the number of darts thrown at the dartboard. To verify this, we write a short function that, given the number of darts to throw, n, returns an estimate of π.

```
> estimate.pi <- function(n=1000) {
+   ## Return an estimate of PI using dartboard method
+   ## with N trials.
+   r <- 1                             # radius of dartboard
+   x <- runif(n, min=-r, max=r)
+   y <- runif(n, min=-r, max=r)
+   l <- sqrt(x^2 + y^2)
+   d <- length(which(l<r))
+   4*d/n
+ }
```

We can then test the procedure a few times, using the default number of darts, 1000:

```
> replicate(9, estimate.pi())

[1] 3.132 3.164 3.180 3.164 3.088 3.048 3.116 3.112 3.140
```

Finally, for a given value of n, we can show 99 estimates of π, as clearly the estimate will vary from run to run. In the following plot, we compare the estimates of π for three different values of n:

```
> ns <- 10^c(2,3,4)
> res <- lapply(ns, function(n) replicate(99, estimate.pi(n)))
> par(las=1, bty='n')
> stripchart(res, method="jitter", group.names=ns,
+            xlab="number of darts",
+            ylab=expression(paste('estimate of ', pi)),
+            vert=T, pch=20, cex=0.5)
> abline(h=pi, col='red')
```

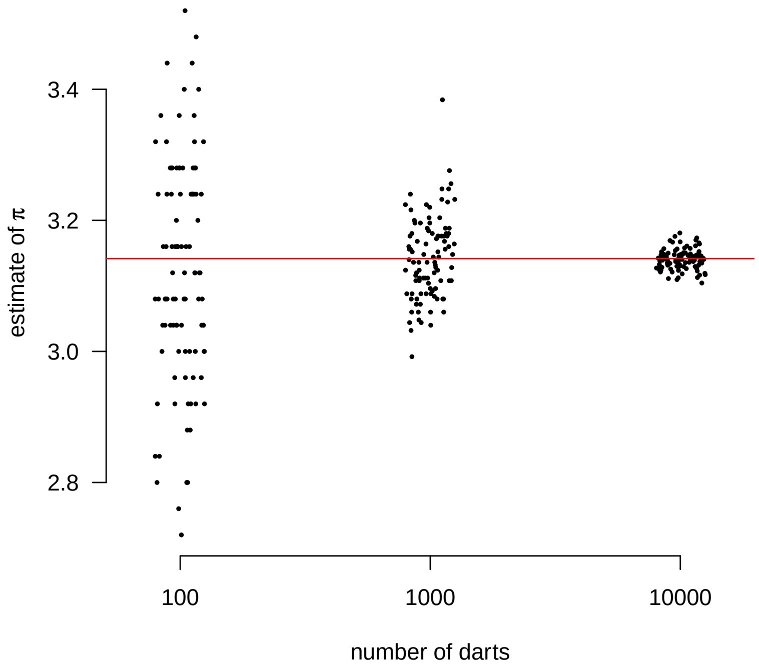

As the number of darts increases, the estimate of π gradually converges onto the actual value of π (shown by the solid red line).
